# Physical activity and social interaction assessments in schoolyard settings using the System for Observing Outdoor Play Environments in Neighborhood Schools (SOOPEN)

**DOI:** 10.1186/s12966-023-01483-5

**Published:** 2023-08-01

**Authors:** Marnie F. Hazlehurst, Kathleen L. Wolf, Cary Simmons, Carolina Nieto, Mary Kathleen Steiner, Kimberly A. Garrett, Anna V. Faino, Mònica Ubalde López, María López-Toribio, Pooja S. Tandon

**Affiliations:** 1 Department of Environmental and Occupational Health Sciences, University of Washington School of Public Health, Seattle, WA USA; 2 College of the Environment, University of Washington, Seattle, WA USA; 3 The Trust for Public Land, Washington DC, USA; 4 Seattle Children’s Research Institute, Seattle, WA USA; 5 Barcelona Institute for Global Health-ISGlobal, Barcelona, Spain; 6 Center for Biomedical Research Network in Epidemiology and Public Health (CIBERESP), Madrid, Spain; 7 University Pompeu Fabra, Barcelona, Spain; 8 Department of Preventive Medicine and Epidemiology, Hospital Clínic of Barcelona, Barcelona, Spain; 9 Department of Pediatrics, University of Washington, Seattle, WA USA

**Keywords:** Physical activity, Prosocial behavior, Recess, Schoolyard

## Abstract

**Background:**

The schoolyard environment provides key opportunities to promote physical activity and socioemotional development for children. Schoolyards can also serve as a community park resource outside of school hours. We aimed to: (i) implement and evaluate reliability of the System for Observing Outdoor Play Environments in Neighborhood Schools (SOOPEN), (ii) assess schoolyard use by children during recess and community members of all ages outside of school hours, and (iii) investigate relationships of schoolyard and children´s group characteristics with physical activity levels and prosocial interactions.

**Methods:**

In this cross-sectional study, we observed student and community visitor behavior using SOOPEN at three urban elementary schoolyards in Tacoma, Washington, USA, prior to renovations intended to expand each facility’s use as a community park in neighborhoods with poor park access. We assessed interrater reliability using intraclass correlation coefficients and described current levels of schoolyard use (at the group level), physical activity, and prosocial behavior. Physical activity was assessed on a five-point scale and dichotomized to indicate moderate-to-vigorous physical activity (MVPA). Social interactions were coded as prosocial, antisocial, or neutral. We examined associations of selected schoolyard features and group characteristics with group MVPA and prosocial behavior during recess using modified Poisson regression to estimate prevalence ratios (PR) and 95% confidence intervals (CI).

**Results:**

We observed a total of 981 activity-defined, informal groups in the schoolyards, and achieved good to excellent interrater reliability using SOOPEN. Community use of the schoolyards during evenings and weekends was limited (n = 56 groups). During 26, 25–50 min recess periods (n = 833 groups), 19% of groups were engaged in MVPA. Schoolyard areas with paved surfaces were associated with more MVPA (PR = 1.52, 95% CI: 1.04, 2.23) compared to field/grass areas; supervised groups were associated with less MVPA than groups not directly supervised by an adult (PR = 0.59, 95% CI: 0.36, 0.96). Schoolyard characteristics were not associated with prosocial behavior. Mixed-gender groups were associated with more MVPA and more prosocial behavior.

**Conclusions:**

Our study using SOOPEN, a reliable new activity observation tool, highlights the multi-dimensional dynamics of physical activity and social interactions in schoolyards, which could be leveraged to promote healthy behaviors during and outside of school hours.

**Supplementary Information:**

The online version contains supplementary material available at 10.1186/s12966-023-01483-5.

## Background

Physical activity (PA) is a key, potentially modifiable, pathway to decreasing health disparities and promoting health and psychosocial resilience in youth [1, 2]. PA is associated with both physical and psychological health benefits for children, including improved cognition and reduced risk of childhood obesity and depression [3]. While current guidelines recommend that school-age children (ages 6 and up) engage in at least 60 min of moderate-to-vigorous physical activity (MVPA) per day [1], the majority of children in the USA do not currently meet these guidelines [4]. Given the amount of time children spend in school and because PA can promote health and learning [5], a whole-of-school approach has been recommended to equitably support students in attaining adequate daily PA. Schoolyards are a critical spatial resource for PA and play, both associated with socioemotional development in children and linked to health outcomes in childhood and later in life. The American Academy of Pediatrics supports recess and play as important for peer interactions and for acquiring lifelong skills of communication, cooperation, and coping as foundations for healthy development [6].

The physical environmental features of the schoolyard and recess programming, such as adult supervision and adult-led activities, as well as child characteristics, may contribute to children’s PA and social interactions [7–9]. Prior studies of recess interventions suggest that portable equipment such as balls and jump ropes, playground markings, and playground markings associated with physical structures increase children’s PA during recess times [10, 11]. The role of supervising adults in associations with children’s PA during recess has been mixed. While some have observed no relationship between the number of supervising adults and MVPA [12], others have identified increases in PA when teachers introduce organized games and attempt to engage students in PA [13]. Access to greenspace may also promote PA during recess; studies of greening interventions suggest that adding natural elements and greenspace to the schoolyard is associated with increases in MVPA, particularly for girls [14–16]. Evidence is more mixed for behavioral outcomes, though some suggest a decrease in verbal conflict after a schoolyard greening intervention [14]. Evidence from observational studies indicates that greenspace at or around the school location is beneficially associated with several children’s outcomes, including higher prosocial behavior and fewer problem behaviors, which may contribute to child health and academic success [6, 17–19].

Several approaches have been used to assess PA and behavior in the schoolyard, including direct observation, self-report questionnaires completed by students, parents, and/or teachers, and accelerometry [16]. A new direct observation tool, the System for Observing Outdoor Play Environments in Neighborhood Schools (SOOPEN), has been developed to evaluate social interactions and PA in schoolyards, with a focus on assessment of group level dynamics [20].

While schoolyards are typically used primarily for recess and afterschool programs, they can also serve as community resources outside of school hours. Green schoolyard redesign and reprogramming are strategies to potentially improve neighborhood access to green space, especially in communities with inadequate access to other park spaces and where space may be limited [21, 22]. A green schoolyards intervention is underway as part of a Trust for Public Land (TPL) national Parks and Community Schoolyards initiative [23, 24]. The goal of this study is to address several research questions using baseline data collected prior to future schoolyard renovations. Specifically, our aims were threefold: (1) to report on the implementation procedures and reliability of SOOPEN; (2) to describe current levels of schoolyard use, PA levels, and prosocial behavior by (a) children during recess, (b) children and families directly after school, and (c) community members during evenings and weekends; and (3) to examine whether PA levels and prosocial behavior vary by schoolyard or group characteristics.

## Methods

### Location and context

Data were collected as part of baseline observations for a study evaluating schoolyard renovations in Tacoma, Washington, USA. Elementary schoolyards located in neighborhoods having the lowest rates of park access in Washington State were selected for extensive redesign in collaboration with local partners. Two schools are undergoing the redesign and rebuild process and a third was selected as a control site by matching on demographics. While future work will take advantage of this quasi-experiment, in the present study we included baseline data (i.e. pre-renovation) from all three schools (kindergarten-grade 5, ages 5–10). This study was approved by both Seattle Children’s Hospital *(STUDY00002677)* and University of Washington *(STUDY00011253)* Institutional Review Boards and determined to be exempt. School leadership agreed to have data collectors from the research team on campus for data collection.

### SOOPEN implementation

We conducted observations at each of three elementary schools during spring and summer of 2022 using SOOPEN. SOOPEN was recently developed to evaluate PA and social behavior at the group level during school recess, rather than the individual level used by prior tools [20, 25]. SOOPEN was developed based on prior direct observation methods: the System for Observing Play and Leisure Activity in Youth (SOPLAY) which assesses PA at the individual level, and the System for Observing Children’s Activity and Relationships during Play (SOCARP), which assesses both PA and social behavior at the individual level.

**Fig. 1:**
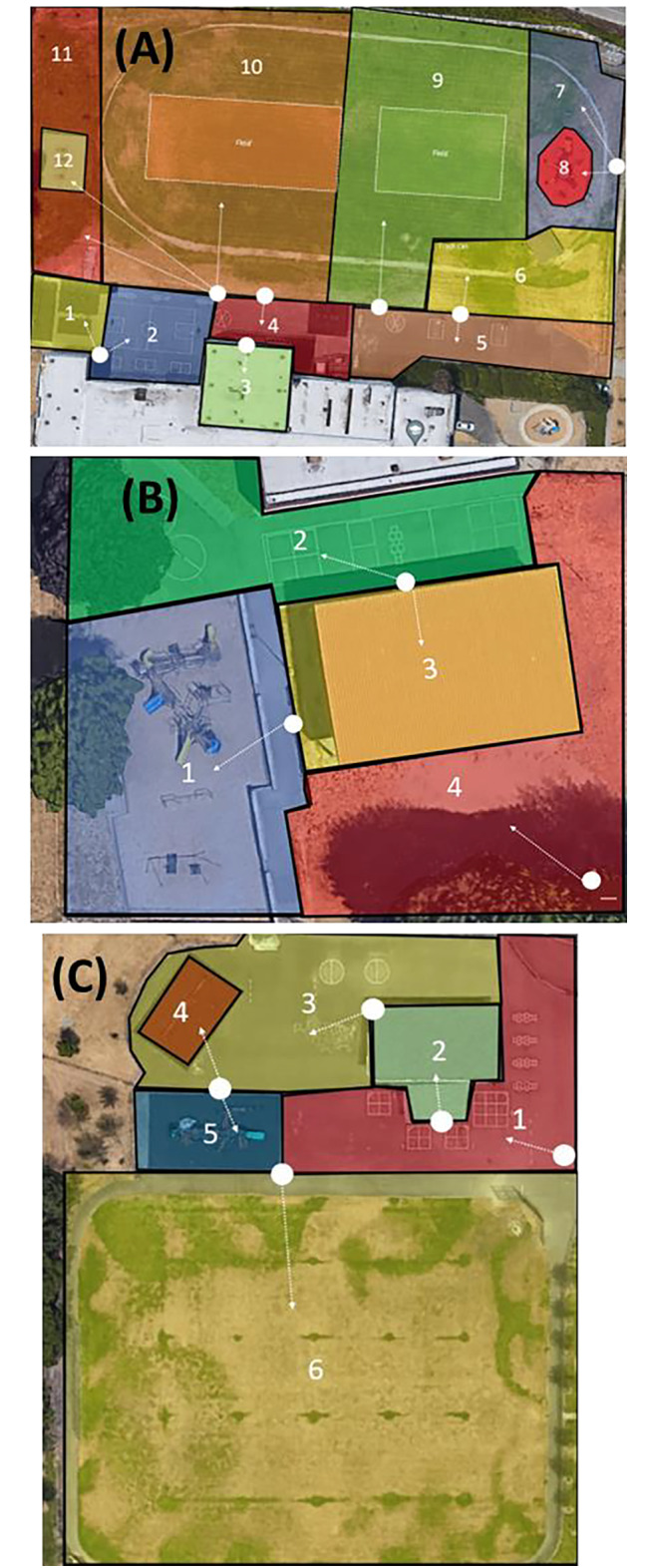
**(a)** Schoolyard 1, **(b)** schoolyard 2, and **(c)** schoolyard 3 target areas for SOOPEN observations. Black outlines indicate the boundaries of each target area and white dots indicate the location where observers stood to conduct scans. For example, at School 3 (panel C), zone 1 is a paved surface, zone 2 is a covered basketball court (paved surface), zone 3 is a paved surface, zone 4 is a set of swings, zone 5 is a play structure, and zone 6 is a grass field bordered by a paved walking path

Similar to SOPLAY, SOOPEN is based on momentary time sampling techniques in which observers conduct systematic scans of pre-specified target areas across the study site [20]. Target areas in each schoolyard were delimited prior to an observation session by the project coordinator according to SOOPEN protocol. Each target area was determined based on identification of areas having specific boundaries, such as schoolyard playground markings or permanent structures, and a single primary feature within those boundaries, such as a play structure, basketball court, or field (Fig. 1). Each target area was classified as a type of schoolyard zone based on the primary feature: paved surfaces, field/grass, or play structure/swings. Large areas or areas with fast moving activities and many groups of students were subdivided into multiple target areas. Within each target area a location was identified whereby the observers could stand and scan the entire target area without any visual obstruction. Each period of time during which observers were recording data for a given target area was considered a scan; multiple groups were observed per scan.

SOOPEN observers recorded contextual factors for each scan of a target area, including weather, time of day and the following characteristics of each target area: type (paved surface, field/grass, or play structure/swings), accessibility, usability, direct adult supervision, presence of organized PA, presence and type of portable equipment provided by the school, and presence of shade. SOOPEN observers also recorded data for each group of individuals within the target area during the scan. Use of the schoolyard was assessed based on the number of groups observed. Observations of all groups of individuals within the target area included characteristics such as group size (alone, small [2–4 individuals], medium [5–9 individuals], or large [10 + individuals]) and perceived gender (count of boys and girls) as well as assessments of PA levels and social behaviors. Additional details on the type of activity a group was engaged in were also noted, such as an organized sports game.

PA was assessed as a five-level scale (1 = Lying down, 2 = Sitting, 3 = Standing, 4 = Walking, 5 = Vigorous) in SOCARP. Validation studies using accelerometry have found the PA scale in the SOCARP tool to be valid for assessing PA at the individual level [26]. In SOOPEN, PA is assessed with the same scale, recording the level exhibited by the majority of the individuals within a group at a specific moment. For descriptive purposes and for comparison with other studies, categories 1–3 were combined and considered sedentary. Moderate to vigorous physical activity (MVPA) was defined by dichotomizing the SOOPEN PA scale; consistent with prior studies using the same 5-point scale, a raw value of 5 was considered MVPA. As a secondary outcome, we also considered a combined MVPA and walking outcome, defined as a raw value of either 4 or 5 on the SOOPEN PA scale in comparison to categories 1–3. While some walking may not be sufficiently active to contribute to a child’s daily recommendation for 60 min of MVPA, brisk walking that elevates the heartrate may contribute to MVPA and thus combined MVPA and walking was examined as a secondary outcome here.

For social interactions, SOOPEN observers record social behaviors of each group in five categories: none, physical pro-social behaviors, non-physical pro-social behaviors, physical conflict, and non-physical conflict [20]. Pro-social behaviors are those characterized by positive social interactions among individuals in the group, including providing support, assistance, feedback, or explanation, whereas antisocial behaviors include negative or threatening social interactions based on either the tone or content of the interaction [20]. Each of these categories is further divided into physical and non-physical behaviors. For each group observed, the interaction recorded reflects the behavior of the majority of that group. Verbal and physical prosocial behavior were combined for our primary binary outcome of prosocial behavior and compared to all other behavior (none or conflict).

Implementation of the SOOPEN tool was adapted to our particular research circumstances. The modified observation form is included in the Supplementary Material (Supplemental Fig. 1). The tool was designed for observations conducted during recess periods, and we extended this use to conduct observations directly after school and during evenings and weekends. A group age variable (approximate age of individuals present categorized as children, teens, adults, seniors, or any combination of those groups) was added to the observation form to provide information on who was using these schoolyards during times outside of school hours. Perceived gender was recorded at the group level (female-only group, male-only group, or mixed gender group), rather than as counts of individuals. Additional contextual information was slightly modified; rather than assessing the presence or absence of shade, we specified the type of coverage-built structure providing cover versus tree canopy, in addition to indicating lack of any cover, for the specific location the group was observed. Additional activity codes, “gardening/engaging with nature” and “didactic activity led by adult”, were also added, given the overall project’s focus on nature contact.

Observers completed two trainings prior to data collection, including a 1-hour online portion and a 2-hour site-based portion. During all recess sessions and most sessions outside of school hours, data were collected by two observers. After all data collection was completed and prior to any data analysis, one observer was designated as primary for each observation period; observers did not know whether their observations would be considered as primary or secondary while they were completing data collection. Each school was observed during two recess periods, two afterschool periods, two evenings, and two weekend days (Supplemental Fig. 2). Recess observations were completed during two days at each school in the 2022 spring season and lasted the full length of all school recess periods on those days. The number of recess periods per day [2–6] and recess duration (25–50 min) varied across the three schools. Each target area in the schoolyard was scanned once during each recess period; the total duration of each scan was calculated as the length of the recess period divided by the number of target areas to be scanned during that recess period. Afterschool and evening observation periods were conducted for one hour each on two separate weekdays. On weekend days we conducted four 1-hour observations (morning, early afternoon, late afternoon, and evening) based on the System for Observing Play and Recreation in Communities (SOPARC) protocol guidelines [27, 28]. Observations for evenings and weekends were conducted in both spring and summer and each target area was scanned once during the 1-hour observation period.

### Statistical analysis

Descriptive analysis included use of the park both during recess hours and outside of school hours as well as characteristics of both scans of the target areas and the observed groups, and are reported as observation frequencies and proportions or means with standard deviations.

Data only from the primary observer was used for analyses. Inter-rater reliability of the SOOPEN in this study using data from both observers (i.e. raters) was assessed using intraclass correlation coefficients (ICCs). One-way consistency ICCs were calculated for the total number of groups observed, the PA scale, and the behavioral scale using data from scans that were conducted by two observers. Specifically, the ICC was calculated for paired observations within a given target area during each observation shift (identified by date, time, and school). ICCs were calculated within each PA level and each behavioral category. For example, if rater 1 had 4 different groups for a specific time, day, and area, and rated one of the groups as sedentary for activity level, then the proportion for sedentary activity level was 0.25. All ICCs were also reported for recess and other observation periods (afterschool, evenings, and weekends combined) separately.

Due to the small number of groups during afterschool, evening, and weekend observation periods, regression models were only feasible for data collected during recess. Only data from the primary observer was used in regression models. The two primary outcomes of interest were MVPA (defined as yes/no in highest PA category for each group observed) and prosocial behavior (defined as yes/no for either verbal or physical prosocial behavior observed for each group). We fit Poisson regression models with robust standard errors (modified Poisson) to estimate prevalence ratios (PRs) and 95% confidence intervals (CI) for associations with MVPA and prosocial behavior. In a secondary analysis, we examined PA defined as either walking or MVPA, similarly using a modified Poisson regression model. All models included the following characteristics: type of schoolyard zone (field/grass, paved surfaces, or play structure/swings), covered area (none, built cover, or tree canopy), portable equipment available (yes/ no), direct adult supervision (yes/no), and group gender (female-only, male-only, or mixed-gender). Due to the small number of schools in this study (n = 3), our ability to estimate between-school variability using a random effect was limited; to account for potential confounding by school-level factors, all models included a fixed effect for school. Analyses were conducted using R 4.1 (R Foundation for Statistical Computing, Vienna, Austria).

## Results

### Inter-rater reliability

In this study, observers conducted 424 primary scans of 22 target areas. Of the 424 primary scans completed, 357 (89%) were also conducted by a second observer and were included in assessments of inter-rater reliability of SOOPEN in this setting. Scans with zero groups observed (n = 209) were excluded for the purpose of calculating ICCs for PA and social interactions. Table 1 reports the ICC for all observations combined, as well as separately for recess, afterschool, and evenings/weekends; overall reliability was good to excellent [29]. Agreement between raters for the number of groups recorded during each scan was high (ICC = 0.992). In general, the ICC was also high when considering the proportion of groups in each PA category, with the exception of walking during evening/weekend observation periods when there were few observations (ICC = 0.427). The ICC was generally lower for prosocial interactions and for verbal categories (both conflict and pro-social) than for PA. The ICC tended to be slightly higher during afterschool observation periods, compared with either recess or evenings/weekends.

**Table 1:** Intraclass Correlation Coefficients of SOOPEN observations of groups, physical activity, and social interactions

	Overall		Recess		Afterschool		Evenings and Weekends	
	*ICC*	*(95% CI)*	*ICC*	*(95% CI)*	*ICC*	*(95% CI)*	*ICC*	*(95% CI)*
**Number of groups**	0.992	(0.989–0.994)	0.960	(0.930, 0.977)	0.964	(0.911, 0.986)	0.785	(0.602, 0.890)
**Physical Activity**								
Sedentary	0.931	(0.898–0.954)	0.897	(0.827–0.940)	0.896	(0.755, 0.958)	0.841	(0.699, 0.920)
Walking	0.906	(0.861–0.937)	0.851	(0.753–0.913)	0.774	(0.509, 0.906)	0.427	(0.095, 0.674)
Vigorous	0.884	(0.829–0.922)	0.830	(0.719–0.899)	0.848	(0.653, 0.938)	0.929	(0.860, 0.965)
**Social Interactions**								
Conflict, Verbal	0.707	(0.587–0.796)	0.696	(0.522–0.815)	-	-	-	-
Conflict, Physical	0.961	(0.942–0.974)	0.959	(0.929–0.976)	-	-	-	-
None	0.936	(0.904–0.957)	0.850	(0.750–0.912)	0.797	(0.554, 0.916)	0.701	(0.469, 0.843)
Prosocial, Physical	0.872	(0.812–0.913)	0.735	(0.577–0.840)	0.968	(0.921, 0.988)	0.688	(0.448, 0.835)
Prosocial, Verbal	0.842	(0.771–0.893)	0.702	(0.530–0.819)	0.920	(0.807, 0.968)	0.618	(0.346, 0.795)

Abbreviations: ICC = Intraclass Correlation Coefficient; 95% CI = 95% confidence interval.

### Descriptive statistics

A total of 22 target areas across three schools were identified for SOOPEN observation: 9 target areas with paved surfaces, 7 target areas identified as field/grass areas, and 6 areas where the primary feature was a play structure or swings. Observers conducted a total of 424 primary scans across these target areas; 160 (38%) of these scans occurred during recess periods (Table 2). The weather was generally mild during SOOPEN observations, with most observations in May completed on cloudy days with an average temperature of 12.2 ˚C during recess and 15.6 ˚C during afterschool periods. Average temperature during evening and weekend observations was slightly higher (18.9 ˚C) due to additional observation days conducted during August. Table 2 shows the characteristics of the schoolyards during the observation periods. Portable equipment (e.g. basketball, soccer ball, jump rope) was available within the target area during 71% of scans during recess and 91% of those scans indicated direct adult supervision within the same target area. In contrast, equipment was available in a smaller proportion of scans during afterschool or evenings and weekends (9% and 6%, respectively), and direct adult supervision was lower during these periods (25% and 3%, respectively) than during recess. Few organized physical activities such as youth league games or competitions were observed in these schoolyards during any scans. Many scans during the afterschool periods and evenings and weekends did not observe any groups (57% and 86%, respectively).

**Table 2:** Schoolyard use and characteristics of SOOPEN scans

	Recess	Afterschool	Evenings and Weekends
**Total Number of Scans**	160	44	220
**Target Area (N/%)**			
Field or grass	42 (26%)	14 (32%)	70 (32%)
Paved surfaces	72 (45%)	18 (41%)	90 (41%)
Play structure or swings	46 (29%)	12 (27%)	60 (27%)
**Portable equipment available (N/%)**			
Yes	114 (71%)	4 (9%)	13 (6%)
No	47 (29%)	40 (91%)	207 (94%)
**Direct supervision (N/%)**			
Yes	146 (91%)	11 (25%)	6 (3%)
No	14 (9%)	33 (75%)	213 (97%)
**Organized physical activity (N/%)**			
Yes	2 (1%)	2 (5%)	0 (0%)
No	158 (99%)	41 (95%)	219 (100%)
**Observed Groups (N/%)**			
0	4 (2%)	25 (57%)	189 (86%)
1 or more	156 (98%)	19 (43%)	31 (14%)
**Groups per scan (Mean/SD)**	5.2 (3.7)	2.1 (3.7)	0.3 (0.7)

The majority of schoolyard use was observed during recess (n = 833 groups, 85% of groups observed during all observation times), with 94% of scans during recess observing at least one group; characteristics of observed groups are shown in Table 3. Most groups were small, either a single individual or a group of 2–4 individuals, and tended to be either all female (42%) or all male (34%) rather than mixed-gender groups (24%). During recess, 19% of groups were rated as engaging in moderate to vigorous activity, 45% as walking, and 36% as sedentary. There were 19 groups (2.3%) who were coded as gardening or interacting with nature during recess (79% of which were observed in target areas classified as grass/field). More frequent activity codes during recess times included locomotion (running or walking not as part of an organized game, 39%) and playing sports (9%) or other active games (6%). Social interactions were most often prosocial (22% physical, 35% verbal), followed by observations with no interactions (40%), and with few conflict interactions observed.

**Table 3:** Characteristics of observed groups using schoolyards

	Recess	Afterschool	Evenings and Weekends
**Total number of groups**	833	92	56
**Group location (N/%)**			
Field or grass	251 (30%)	12 (13%)	5 (9%)
Paved surfaces	304 (37%)	49 (53%)	32 (57%)
Play structure or swings	278 (33%)	31 (34%)	19 (34%)
**Group size (N/%)**			
Alone (1)	317 (38%)	42 (46%)	18 (32%)
Small (2–4)	420 (50%)	47 (51%)	36 (64%)
Medium (5–9)	68 (8%)	2 (2%)	2 (4%)
Large (10+)	28 (3%)	1 (1%)	0 (0%)
**Age Group (N/%)**			
Children only	802 (96%)	58 (63%)	22 (40%)
Children and teens	7 (1%)	1 (1%)	1 (2%)
Children and adults	24 (3%)	18 (20%)	17 (30%)
Children, teens, and adults	0 (0%)	0 (0%)	1 (2%)
Teens only	0 (0%)	1 (1%)	3 (5%)
Teens and adults	0 (0%)	0 (0%)	2 (4%)
Adults only	0 (0%)	14 (15%)	8 (14%)
Seniors only	0 (0%)	0 (0%)	2 (4%)
**Gender (N/%)**			
Female	349 (42%)	41 (45%)	18 (32%)
Male	281 (34%)	32 (35%)	21 (38%)
Both	198 (24%)	19 (21%)	17 (30%)
**Activity Level (N/%)**			
Sedentary	295 (36%)	22 (24%)	23 (42%)
Walking	376 (45%)	49 (53%)	28 (51%)
Moderate/Vigorous	159 (19%)	21 (23%)	4 (7%)
**Social Interactions (N/%)**			
None	335 (40%)	47 (51%)	21 (38%)
Prosocial, verbal	288 (35%)	25 (27%)	25 (45%)
Prosocial, physical	183 (22%)	20 (22%)	10 (18%)
Conflict, verbal	7 (1%)	0 (0%)	0 (0%)
Conflict, physical	12 (1%)	0 (0%)	0 (0%)
**Activity Code (N/%)** ^**a**^			
Sports	71 (9%)	6 (7%)	7 (13%)
Active games	50 (6%)	2 (2%)	2 (4%)
Locomotion	328 (39%)	46 (50%)	19 (34%)
Interacting with nature/gardening	19 (2%)	0 (0%)	0 (0%)
Didactic activity	1 (0.1%)	0 (0%)	0 (0%)
Sedentary activity	349 (42%)	33 (34%)	26 (46%)

^a^More than one activity could be assigned to a single group, so numbers add to greater than the total count of groups and greater than 100%. Sports included football, basketball, or other sport-related activities. Active games included organized physical activity not categorized as a sport such as some throwing and catching games, hopscotch, four-square, chasing games/tag, jump rope, etc. Locomotion activity codes were noted when children were running or walking when that activity that was not part of a sport or active game (e.g. in transition). Sedentary activity codes included eating, talking, reading/writing/artwork, board games, and viewing others’ games as a spectator

Schoolyard use was also observed during the afterschool window, with 43% of scans including at least one group. The highest proportion of MVPA (23%) and lowest proportion of sedentary activity (24%) was observed in this time period compared to recess and evenings/weekends. No interaction with nature activity codes were recorded during afterschool scans.

Despite the large number of scans conducted on evenings and weekends (n = 220), groups using the schoolyard (n = 56 groups) were observed in only 15% of scans. More variability in group size and age was observed during evenings and weekends. Only 7% of groups were engaging in MVPA while 42% were observed in sedentary activities; 45% of groups had verbal prosocial interactions. No conflict interactions were observed.

### Physical activity (PA)

Several associations were observed in models of MVPA (Table 4). Schoolyard zone was associated with MVPA; paved surfaces were associated with a 52% higher prevalence of MVPA (95% CI: 1.04–2.23) and play structure/swing zones were associated with a 42% lower prevalence of MVPA (95% CI: 0.35–0.95) compared to field/grass zones. Covered areas and availability of portable equipment were not associated with MVPA during recess. Direct supervision within the target area was associated with a 41% lower prevalence of MVPA compared to no direct supervision (95% CI: 0.36–0.96). Additionally, there was some suggestion that single-gender groups had a 32% lower prevalence of MVPA compared to mixed-gender groups (PR = 0.68, 95% CI: 0.48–0.96 and PR = 0.68, 95% CI: 0.48–0.95 for male-only and female-only groups, respectively).

Similarly, schoolyard zone was associated with combined PA including MVPA and walking, such that less PA was observed on play structure/swing zones compared to field/grass zones (PR = 0.71, 95% CI: 0.60–0.83). Groups under tree cover or built cover were also associated with 45% (95% CI: 0.30–0.98) and 20% (95% CI: 0.68–0.95) lower prevalence, respectively, of the combined PA measure compared to those in areas without any cover. Availability of portable equipment, supervision, and group gender were not associated with this combined activity outcome.

**Table 4:** Cross-sectional associations of schoolyard features and group characteristics with physical activity levels during recess

	Physical Activity Levels during Recess			
	MVPA		Combined MVPA and walking	
	*PR (95% CI)*	*p-value*	*PR (95% CI)*	*p-value*
**Schoolyard Zone**		< 0.001		< 0.001
Field or grass	1 (reference)		1 (reference)	
Paved surfaces	1.52 (1.04, 2.23)		1.10 (0.97, 1.25)	
Play structure or swings	0.58 (0.35, 0.95)		0.71 (0.60, 0.83)	
**Covered Area**		0.150		0.006
No cover	1 (reference)		1 (reference)	
Built cover	0.75 (0.51, 1.11)		0.80 (0.68, 0.95)	
Tree cover	0.39 (0.10, 1.49)		0.55 (0.30, 0.98)	
**Portable Equipment Available**		0.636		0.396
No	1 (reference)		1 (reference)	
Yes	0.92 (0.65, 1.30)		0.95 (0.85, 1.07)	
**Direct Supervision**		0.036		0.828
No	1 (reference)		1 (reference)	
Yes	0.59 (0.36, 0.96)		0.98 (0.85, 1.14)	
**Group Gender**		0.036		0.335
Mixed-gender group	1 (reference)		1 (reference)	
Male-only group	0.68 (0.48, 0.96)		1.03 (0.91, 1.18)	
Female-only group	0.68 (0.48, 0.95)		0.95 (0.83, 1.08)	

^a^All models were additionally adjusted for school as a fixed effect

Abbreviations: MVPA = Moderate-to-vigorous physical activity, PR = prevalence ratio, 95% CI = 95% confidence interval

### Prosocial Behavior

A large proportion (57%) of the groups observed exhibited prosocial behavior (either verbal or physical) during recess periods. Features of the schoolyard, including zone, coverage type, portable equipment availability, and supervision, were not associated with prosocial behavior during recess (Table 5). Single-gender groups had a 42% (95% CI: 0.51–0.66) and 41% (95% CI: 0.52–0.67) lower prevalence of prosocial behavior, for male-only and female-only groups respectively, compared to mixed-gender groups .

**Table 5:** Cross-sectional associations of schoolyard features and group characteristics with prosocial behavior during recess

	Prosocial Interactions during Recess	
	*PR (95% CI)*	*p-value*
**Schoolyard Zone**		0.256
Field or grass	1 (reference)	
Paved surfaces	0.91 (0.78, 1.06)	
Play structure or swings	0.88 (0.75, 1.03)	
**Covered Area**		0.857
No cover	1 (reference)	
Built cover	1.03 (0.86, 1.24)	
Tree cover	1.09 (0.75, 1.58)	
**Portable Equipment Available**		0.451
No	1 (reference)	
Yes	0.95 (0.83, 1.09)	
**Direct Supervision**		0.179
No	1 (reference)	
Yes	1.17 (0.93, 1.47)	
**Group Gender**		< 0.001
Mixed-gender group	1 (reference)	
Male-only group	0.58 (0.51, 0.66)	
Female-only group	0.59 (0.52, 0.67)	

^a^All models were additionally adjusted for school as a fixed effect

Abbreviations: PR = prevalence ratio, 95% CI = 95% confidence interval

## Discussion

Our study adds to prior literature examining cross-sectional relationships of multi-level factors with PA and prosocial behavior in schoolyards. The SOOPEN tool was successfully implemented in our study, with good to excellent inter-rater reliability for observations of group PA and social interactions. We found that during recess, MVPA was higher in schoolyard zones with paved surfaces compared to grass areas, for groups not directly supervised compared to directly supervised groups, and for mixed-gender groups compared to single-gender groups. While schoolyard characteristics did not appear to be associated with prosocial behavior, mixed-gender groups were estimated to have a higher prevalence of prosocial behavior during recess. Additionally, we found that community use of schoolyards was quite low during evenings and weekends and this small sample size prevented further analysis of groups during those times.

Physical features of urban parks and playgrounds may promote or discourage different PA levels and types of activities. For example, greenspace in neighborhood parks has been linked to increased PA across multiple age groups; higher levels of PA have been associated with larger greenspace size, the presence of shade trees or forested areas, and elements such as paths and water features [30–33]. However, studies of schoolyards have been more mixed with regards to the role of environmental characteristics such as greenspace in PA promotion. Qualitative and quantitative studies suggest that both sports areas and greenspace are desired features for PA, though these relationships may change across childhood with sports facilities becoming more important for MVPA as children reach school-age [34]. A study of schoolyards in Denmark found the highest proportion of time in MVPA occurred in grass (27%) and playground areas (26%) compared to multi-court areas (22%) [35]. However, others did not find associations between schoolyard characteristics and PA levels [36, 37]. In our study, we observed higher levels of activity in paved target areas. Additionally, much of the portable equipment (e.g. basketballs and jump ropes), was placed in and specifically for use in those paved areas. Our findings suggest that paved surfaces should be considered when planning or re-designing schoolyards to support more active play, and that community park implementation could include provisions for equipment access or check-out. However, the paved surfaces at the study schools are currently primarily concrete and asphalt, and use of other surface materials may both provide areas that support children’s PA as well as mitigate environmental hazards (e.g. reducing temperatures, managing stormwater runoff) to create spaces more resilient to climate change effects. Natural elements such as trees and plants can also be incorporated in paved areas. Furthermore, addressing water drainage in grassy areas may make those areas more appealing for children, facilitating active games that both increase levels of PA and promote inclusive, creative play and psychomotricity development for children [16]. Prior studies suggest such areas may especially increase PA among girls [15].

In addition to the physical characteristics of the schoolyard space, programming and organized events within parks or adult-led activity may also influence levels of children’s PA. Several prior studies have found mixed results regarding teacher-initiated activities, with some identifying associations with higher levels of PA but others observing mixed results depending on the subgroup of children (e.g. by age or gender) examined [38–40]. In our study, direct supervision within the target area was associated with a lower prevalence of MVPA compared to no direct supervision, after adjusting for the other variables in the model. It is possible that children without direct supervision engaged in more active, free play perhaps without interference from a nearby adult. Children may also have tended to be in transit when observed in paved, unsupervised areas closer to the school building, slowing down when arriving at their destination within the schoolyard, where supervisors were located. It is also possible that since staffing constraints during recess make it impossible for adult supervisors to have been present in every target area, the adults chose to monitor areas that they deemed potentially more problematic. Perhaps children that had organized themselves into an active game such as basketball or tag, were thought to be less likely to need direct supervision. Also, supervision in this tool does not differentiate between an adult that is simply physically present versus someone encouraging or engaging in active play with children. There is evidence that programs where adults help set up activities during recess are beneficial for promoting safe, inclusive and higher intensity PA, suggesting that the role and actions of the adults present can be impactful [13, 41].

Recess also plays an important role in the socioemotional development for children and the contributions of free play during recess to emotional and social-wellbeing have been increasingly recognized [42]. Specifically, recess has been highlighted as a key time for learning and practicing conflict resolution, cooperation, self-discipline, confidence and communication skills, which are linked with better outcomes in adulthood [43]. In this study, we found a high proportion of prosocial interactions and very low proportion of conflict interactions overall. A cross-sectional study of schoolyards after a renovation found a similarly low proportion of negative interactions (< 3%) but smaller proportion positive interactions (27%) than the 57% observed in our study [44]. Although we observed higher prevalence of prosocial behavior among mixed-gender groups, others did not find individual-level associations between gender and prosocial interactions on the playground [44]. More work is needed to further understand the role of group-level gender in relation to prosocial behavior during recess, including consideration of additional potential confounders such as the social emotional learning curricula provided in the study school district.

An important finding of this study was the identification of sparse use of the schoolyards by community members outside of school hours, especially during evenings and weekends. Though the schoolyards are open to the public and observers were able to access the schoolyards during these times, schoolyard entrances were not always easy to find. Despite a lack of access to other park spaces in the neighborhood, community members may not be aware that they can use the schoolyard after school hours. One key goal of the broader TPL schoolyard renovation effort is to increase access to greenspace in urban areas with a lack of existing greenspace resources. PA during the afterschool window on weekdays is an important contribution to overall PA levels among children [45]. Prior studies have indicated increased utilization of park spaces after renovations [46] though the area surrounding the park may also influence park utilization [47]. A review of prior schoolyard interventions focused on greening found that overall use of the schoolyard spaces generally increased from pre-to-post implementation [48]. Increased use of green schoolyards outside of school hours may also facilitate multi-generational co-participation in PA [49]. Of note in our preliminary findings was greater variability in group size and age, fewer MVPA groups than during school day observations, and substantial prosocial verbal interactions. The Tacoma metropolitan parks board is currently collaborating on the schoolyard to park renovations; these findings can inform program development that enhances beneficial uses and promotes greater MVPA by community users.

This study was part of a broader research program addressing equity and parks. Despite the evidence of health benefits from parks and greenspace exposure, inequities exist in access to these resources. In the US, it is estimated that 100 million people do not have a park within a 10-minute walk of their home [50]. Residents of low-income neighborhoods and neighborhoods where residents predominantly identify as people of color often have access to less park acreage or lower quality parks than residents in high-income or predominantly white neighborhoods [51, 52]. Built environment conditions, including these contemporary patterns of greenspace access, size, and tree canopy have resulted from legacies of discrimination in housing, land use, and urban planning policies that concentrated environmental resources in predominantly white and wealthy communities, while increasing environmental hazards such as major highways in communities of color and in low-income neighborhoods [51, 53, 54]. Schoolyard greening renovations have the potential to address current inequities in park access and increase schoolyard use by community members—a research question for further analysis after renovations are completed.

We acknowledge several limitations in this study. This analysis utilized cross-sectional data from schoolyards within a single USA city, which may limit causal inference as well as generalizability to schoolyards in different climate zones or population densities. The scarcity of groups observed outside of school hours limited our ability to fit regression models for observations outside of recess. The use of direct observations at a single point in time did not allow us to quantify the duration of PA for groups or individuals. Contact with nature and PA in natural settings may provide additional health benefits, but in this study, we were not able to account for other interactions with green features beyond categorizing target areas as field/grass or indicating interaction with nature as an activity code. Upgraded designs of Schools 1 and 2 include enhanced nature elements, providing opportunities for a pre-post study design after construction implementation.

This study also had several strengths. Using systematic direct observation rather than relying on self-report avoids social desirability and recall bias. A contribution of SOOPEN is the added intention of observing group-level social dynamics and PA, as studies show the positive health impacts of park proximity and social engagement [49, 55]. Often, such observation tools focus on the individual physical benefit of PA; however, PA can have emotional and social development benefits, and the desire for social interaction can motivate participation in PA [56]. SOOPEN incorporates this social component in part by utilizing groups as the unit of measurement. Furthermore, compared to individual-monitoring approaches this tool was relatively easy to implement and low cost with good reliability in this setting. We were able to use this tool to conduct observations not only at recess, but outside of school hours as well, broadening the scope of our data and potential applications of SOOPEN.

Prior studies of schoolyard greening have identified increases in children’s PA, particularly among girls, associated with the schoolyard intervention [14, 57]. Some of these studies additionally found an association between schoolyard greening and socioemotional health outcomes [21, 57]. In this study, we observed few groups of children interacting with nature in the schoolyard which is likely driven by few existing opportunities for such interaction. Nature elements in these schoolyards prior to renovation were limited to grassy areas intended for sports activities. Other studies suggest that non-linear shaped grassy areas interspersed with trees and zones with natural features such as rocks and logs promote both higher use of those schoolyard zones by children as well as more PA [7]. Future work after schoolyard renovations can investigate changes in PA, prosocial behavior, and nature contact as well as changes in community use of schoolyard parks associated with the schoolyard greening to further inform both design and programming in schoolyards to promote health behaviors.

## Conclusions

This study aimed to assess the implementation of a new systematic direct observation tool and the results obtained can inform improved measures to understand the public health value of schoolyards that can also serve as community parks. Results can also be used to strategically develop park programming aimed at encouraging specific health outcomes. SOOPEN was a reliable tool for assessing schoolyard use, PA, and social interactions at the group level when implemented during recess. SOOPEN proved feasible to implement beyond recess, but data from outside of school hours in this study was sparse and therefore evaluation of the use of this tool during these time frames is still in development. While high levels of MVPA were observed in paved areas and those areas without direct supervision in the schoolyard prior to renovations, further work is needed to disentangle differences based on programming for physical activity within schoolyard zones and the role of adult-led physical activity, as well as to examine changes after a schoolyard greening intervention. Importantly, few community members were observed using the schoolyard during evenings and weekends; renovations may encourage use of the schoolyards during these times, in neighborhoods lacking access to other park spaces.

## Electronic supplementary material

Below is the link to the electronic supplementary material.

Supplementary Material 1: SOOPEN form and data collection details

## Data Availability

The datasets used and/or analyzed during the current study are available from the corresponding author on reasonable request.

## Abbreviations

PA: Physical activity
SOOPEN: System for Observing Outdoor Play Environments in Neighborhood Schools
ICC: Intraclass correlation coefficient
MVPA: Moderate-to-vigorous physical activity
PR: Prevalence ratio
CI: Confidence interval
SOPLAY: System for Observing Play and Leisure Activity in Youth
SOCARP: System for Observing Children’s Activity and Relationships during Play
GPS: Global Positioning System
USA: United States of America
