# Supplementary material for: Physical activity and social interaction assessments in schoolyard settings using the System for Observing Outdoor Play Environments in Neighborhood Schools (SOOPEN)
Source: Int J Behav Nutr Phys Act. 2023 Aug 1;20:94. doi: 10.1186/s12966-023-01483-5 (PMC10394799; doi:10.1186/s12966-023-01483-5)
Supplement: Supplementary file 1 — Supplementary Material 1: SOOPEN form and data collection details [file 12966_2023_1483_MOESM1_ESM.docx]

**Supplementary Material**

**Physical activity and social interaction assessments in schoolyard settings using the System for Observing Outdoor Play Environments in Neighborhood Schools (SOOPEN)**

Marnie F. Hazlehurst, Kathleen L. Wolf, Cary Simmons, Carolina Nieto, Mary Kathleen Steiner, Kimberly A. Garrett, Anna V. Faino, Mònica Ubalde López, María López-Toribio, Pooja S. Tandon.

**Contents**

**Supplemental Figure 1.** Modified SOOPEN data collection form.

**Supplemental Figure 2.** SOOPEN data collection at three schools in Tacoma, WA.

**Supplemental Figure 2.** Modified SOOPEN data collection form.

**Supplemental Figure 2.** SOOPEN data collection at three schools in Tacoma, WA.


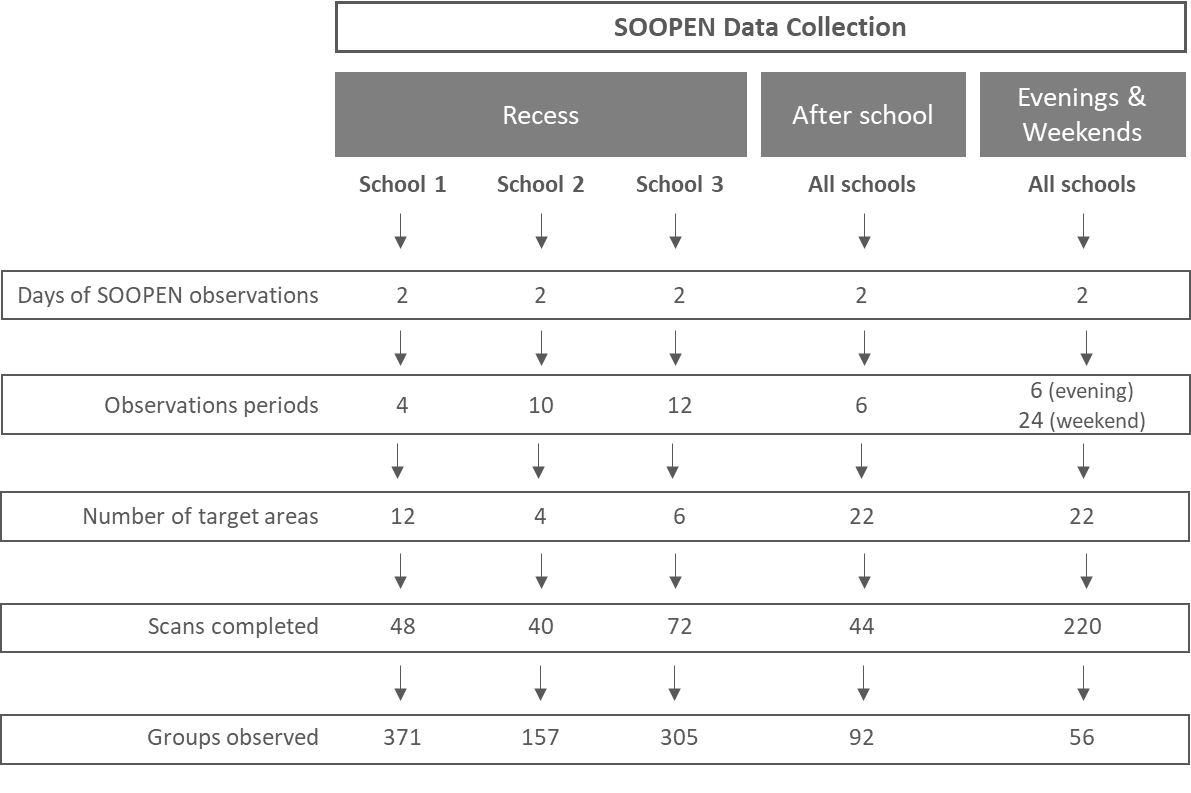


Recess windows are split by school because there were variable number of recess periods during the day. Weekend days were observed at 4 different time points across the day: morning, early afternoon, late afternoon, and evening.
